# Live imaging of individual cell divisions in mouse neuroepithelium shows asymmetry in cilium formation and Sonic hedgehog response

**DOI:** 10.1186/2046-2530-1-6

**Published:** 2012-05-02

**Authors:** Karolina Piotrowska-Nitsche, Tamara Caspary

**Affiliations:** 1 Department of Human Genetics, Emory University School of Medicine, 615 Michael Street, Suite 301, Atlanta, GA 30322, USA; 2 on leave from Department of Experimental Embryology, Polish Academy of Sciences, 05-552 Wolka Kosowska, Poland

**Keywords:** cell division, *ex vivo *live imaging, imaging neuroepithelium, primary cilia, Shh

## Abstract

**Background:**

Primary cilia are microtubule-based sensory organelles that play important roles in developmental signaling pathways. Recent work demonstrated that, in cell culture, the daughter cell that inherits the older mother centriole generates a primary cilium and responds to external stimuli prior to its sister cell. This asynchrony in timing of cilia formation could be especially critical during development as cell divisions are required for both differentiation and maintenance of progenitor cell niches.

**Methods:**

Here we integrate several fluorescent markers and use *ex vivo *live imaging of a single cell division within the mouse E8.5 neuroepithelium to reveal both the formation of a primary cilium and the transcriptional response to Sonic hedgehog in the daughter cells.

**Results:**

We show that, upon cell division, cilia formation and the Sonic hedgehog response are asynchronous between the daughter cells.

**Conclusions:**

Our results demonstrate that we can directly observe single cell divisions within the developing neuroepithelium and concomitantly monitor cilium formation or Sonic hedgehog response. We expect this method to be especially powerful in examining whether cellular behavior can lead to both differentiation and maintenance of cells in a progenitor niche.

## Background

Primary cilia are critical for a number of signaling pathways linked to cell proliferation and differentiation [1-3]. They are often thought of as cellular antennae because they send and receive signals [4-6]. In dividing cells, the cilium must be generated anew after each cell division. The cilium projects from the older centriole of the centrosome, so generation of the cilium is tightly linked to centriole duplication and to the cell cycle [7]. Recent work demonstrated that, in cell culture, the daughter cell that inherits the older mother centriole generates a primary cilium and responds to external stimuli before its sister cell [8]. This asynchrony implies that cell fate may be controlled, in part, by the timing of cilia formation.

**Figure 1:**
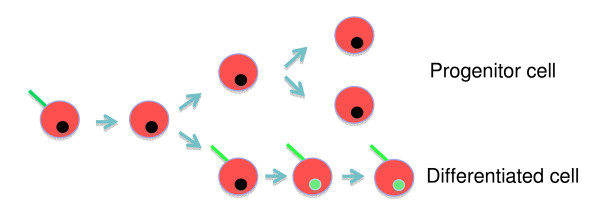
**Cilia asynchrony model**. In a wild type cell, the cilium breaks down prior to cell division. Of the two daughter cells, one forms a cilium before the other, so it is responsive to Shh first (green nucleus) and differentiates; however, the other cell does not become Shh-responsive prior to its subsequent division permitting maintenance of the progenitor niche.

The timing of cilia formation could be especially critical during development as cell divisions are required for both differentiation and maintenance of progenitor cell niches. Most often these distinct fates are physically juxtaposed, raising the question of how cells under such similar environmental cues manage such different responses. Asynchrony in cilium formation offers a potential mechanism. Under such a scenario, when a progenitor divides, one daughter cell forms a cilium and responds to signaling quickly, while the other does not. This would result in an asymmetric division into one differentiated cell and one progenitor cell, which could divide again to maintain the niche (Figure 1).

In this study, we focus on mouse neural tube patterning, specifically on the role of primary cilia in Sonic hedgehog (Shh) signaling [1]. Shh specifies the distinct ventral neural cell fates [9-11]. In order to examine the relative timing of cilia formation and Shh signaling response at a physiological level, we developed a system that integrates live imaging of fluorescent markers in cultured slices of embryonic mouse neuroepithelium. Here we show that this method enables us to trace single cell divisions to assess the relative timing of primary cilia formation and Shh response.

## Methods

### Cell culture

The mouse kidney cell line, IMCD3, stably expressing somatostatin receptor 3 (SsTR3)-GFP in cilia (a kind gift from Greg Pazour) was seeded at low density on the 35 mm glass bottom dish (MatTek, Ashland, MA, USA, part No. P35GC-0-10-C) and grown in DMEM high glucose media without serum at 37°C in 5% CO_2_. After 7.5 hours of serum starvation, cells were cultured in media with 10% FBS at 37°C with 5% CO_2 _during the imaging. Cells were imaged for 15 hours in total. Images were obtained in 15-image z-stack series at 0.4 μm intervals so that 90 images were taken every 10 minutes.

### Whole mouse embryo culture

Embryos at embryonic day E7.5 and E8.5 were dissected in pre-warmed wash medium containing DMEM/F12 (1:1) (GIBCO, Grand Island, NY USA) supplemented with 10% newborn calf serum (Lonza, Lawrenceville, GA USA) and 1% penicillin/streptomycin (Sigma, Saint Louis, MO USA) [12]. Directly after dissection, E8.5 embryos still surrounded by yolk sac were placed on the 37°C heating stage under the fluorescent microscope and identified as GFP and/or dsRed positive. Up to two selected embryos were transferred into a 500-μL drop of pre-equilibrated culture media containing 50% *S*prague-Dawley male rat serum (Harlan Bioproducts, Tampa, FL USA) and 50% DMEM/F12 (1:1) without phenol red supplemented with L-glutamine (GIBCO, Grand Island, NY USA) and 1% of 1 M 4-(2-hydroxyethyl)-1-piperazineethanesulfonic acid in 0.85% NaCl (BioWhittaker, Walkersville, MD USA) and penicillin/streptomycin (Sigma, Saint Louis, MO USA) [12]. A thin layer of equilibrated light mineral oil (Sigma) was placed over the medium to prevent evaporation and the culture dish containing the embryos was transferred to the incubator set at 37°C and 5% CO_2_.

### Live imaging and time-lapse confocal microscopy

Live cell imaging was performed using the Nikon A1R Laser Scanning Confocal Inverted Microscope (Nikon, Tokyo, Japan) equipped with a hybrid scanner that allows for traditional confocal imaging as well as high speed imaging. The excitation wavelengths were 488 and 561 nm. The imaging system was equipped with an environmental chamber that regulates temperature, set to 37°C, and 5% CO_2_. Nikon Perfect Focus was used to ensure absence of focus drift when imaging living cells. The 60× oil-immersion objective was used to record GFP-labeled cilia and dsRed-positive cells, while the 40× oil-immersion objective was used to monitor oligodendrocyte transcription factor 2 (Olig2)-GFP- and dsRed-positive cells. Every 10 minutes we acquired z-stacks of up to 25 μm with spacing of 1.5 μm (40× objective) and up to 8 μm with a spacing of 0.4 μm (60× objective). A separate workstation was equipped with Imaris x64 7.2.3 (Bitplane Inc, South Windsor, CT USA) three-dimensional reconstruction software to analyze recorded data.

### Mice

Mice were cared for according to animal protocols approved by Emory University. Mice used were: Z/RED line (STOCK Tg(CAG-Bgeo,-DsRed*MST)1Nagy/J, Jackson Laboratory, Bar Harbor, ME USA), CAGGCreER™ and the modified bacterial artificial chromosome (BAC) transgenic Olig2-enhanced GFP (eGFP) line (STOCK Tg(Olig2-EGFP)EK23Gsat/Mmcd, MMRRC) which were re-derived [13,14].

### Tamoxifen injection

The tamoxifen was dissolved in 100% ethanol. To initiate recombination, we performed intraperitoneal injections of 2.5 mg tamoxifen (Sigma) per 40 g of body weight into pregnant females at 6.5 days postcoitum. Mouse embryos were collected and analyzed 48 hours post-injection.

### Neural tube slice preparation for live imaging

In order to record dividing neuroepithelial cells at the E8.5 embryonic stage, the neural tube was dissected in pre-warmed wash medium using a micro-knife (Electron Microscopy Sciences, Hatfield, PA USA), size 0.025 mm, on a 1% agar-coated dish. Next, the sample was placed ventral side down in a 150-μL drop of equilibrated culture medium without phenol red on the 35-mm poly-L-lysine coated glass bottom dish (MatTek) and covered with a thin layer of equilibrated light mineral oil (Sigma). To avoid sample movement during imaging, the isolated neural tube was mounted between small amounts of a 1:1 mixture made from 100% pure petroleum jelly and wax, and gently pressed by a narrow piece of glass coverslip in order to immobilize the neural tube.

### Viral infection

Sstr3-GFP lentivirus was packaged and titered by the Emory Viral Core. Approximately two million virions (5 to 10 μL) of virus were added to a 500-μL equilibrated drop of culture medium containing an E8.5 embryo. After culturing at 37°C for 18 hours, the embryo was washed three times in culture media without virus prior to initiating the imaging.

### Immunofluorescence

Embryos were dissected, fixed and frozen, with sections prepared and stained as previously described [15]. Antibodies and their concentrations were: rat monoclonal anti-red fluorescent protein (5F8) 1:200 (Chromotek, San Diego, CA USA); rabbit anti-Arl13b serum 1:1500 and mouse monoclonal anti-Arl13b 1:5 (295B/54, both NeuroMab, University of California, Davis, USA); rabbit anti-Olig2 1:300 (Chemicon, Temecula, CA USA); mouse monoclonals paired box gene 6 (Pax6), Shh and Nkx2.2- all 1:10; (Developmental Hybridoma Bank, Iowa City, IA USA); and rabbit polyclonal Ki67 1:500 (Abcam, Cambridge, MA USA) [16]. Secondary antibodies Alexa Fluor 488, 568 and 350 (Molecular Probes, Eugene, OR USA) to the appropriate species were used at 1:200 concentration. Hoechst 1:3000 (Molecular Probes, Eugene, OR USA) or TO-PRO-3 1:500 (T3605, Molecular Probes, Eugene, OR USA) were used to stain nuclei. Slides were mounted in 80% glycerol and viewed within 24 hours. Images of neural tube sections were collected with a Leica DM6000B upright fluorescence microscope (Leica Microsystems, Inc., Buffalo Grove, Il USA) and processed using the SimplePCI program (Hamamatsu Corporation, Sewickley, PA USA).

### Statistical analysis

We used a paired Student's *t *test to compare the differences between groups. A *P *value < 0.05 was considered statistically significant.

## Results and discussion

### Live imaging of cilia formation in cultured cells

**Figure 2:**
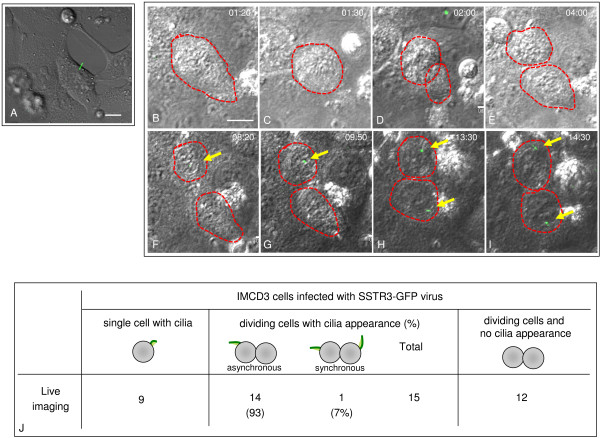
**Somatostatin 3 receptor fused to green fluorescent protein labels cilia in IMCD3 cells**. **(A) **Confocal image of IMCD3 cell expressing SsTR3-GFP in cilia. **(B-I) **Live imaging of a dividing IMCD3 cell with SsTR3-GFP expressed in cilia over 15 hours. Red dashes outline individual cells. Of the two daughter cells, one expresses GFP in cilia ahead of the other daughter cell (F-I; yellow arrow). The associated movie was imaged at a rate of one frame every 10 min. **(J) **Number of IMCD3 cells expressing SsTR3-GFP in cilia during live imaging observation. Of the divisions, 93% resulted in one daughter cell expressing SsTR3-GFP prior to the other daughter cell. Bar is 10 μm (A and B-I).

In order to establish a system whereby we could image cilia formation in real time, we first examined fluorescently labeled cilia in cultured dividing cells. We used a mouse kidney cell line, IMCD3, that stably expresses Sstr3-GFP, a marker of the ciliary membrane (Figure 2A) [17]. We performed time-lapse confocal imaging to observe dividing cells and evaluated cilia formation in their progeny. Over the course of 15 hours of live observation of cell divisions with cilia appearance, we found that 93% of divisions resulted in one daughter cell expressing Sstr3-GFP prior to the other daughter cell (Figure 2B, C, D, E, F, G, H, I, J; Additional file 1). This was similar to previous results in cultured mouse fibroblasts and human epithelial cells [8]. Thus Sstr3-GFP is a suitable marker for live imaging of cilia formation.

### Post-implantation whole mouse embryo culture - *ex vivo *system

**Figure 3:**
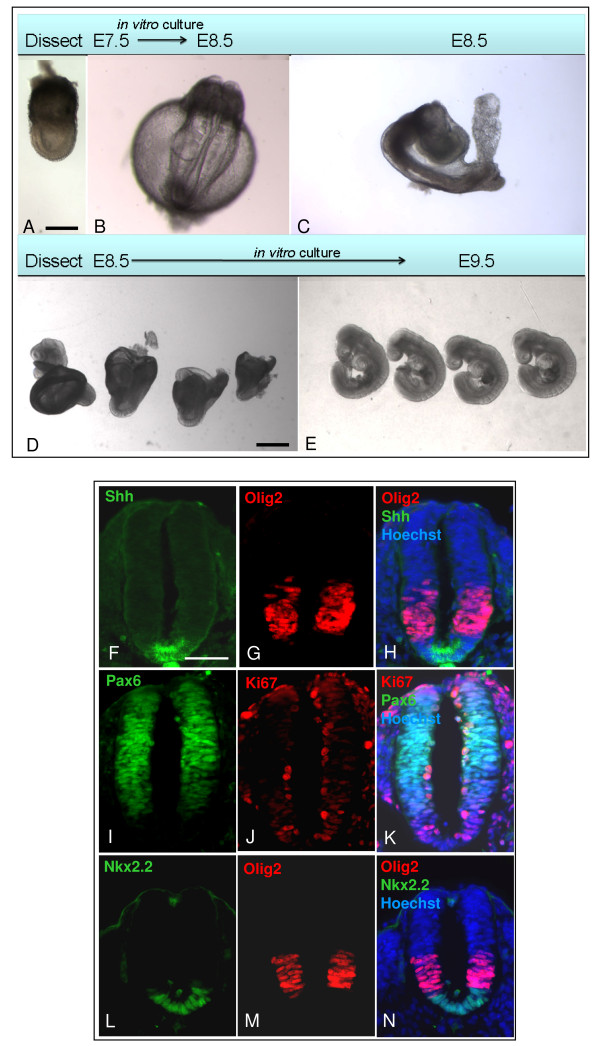
**Development is normal during embryo culture**. **(A) **Wild type embryo collected at E7.5 and cultured *in vitro *for 24 hours develops with **(B-C) **normal morphology with a yolk sac. **(D) **Wild type embryos harvested at E8.5 and **(E) **cultured *in vitro *for 24 hours increase in size with normal developmental milestones including observable heartbeat **(F-N)**. Immunocytochemistry of the wild type embryos fixed at E9.5 after 24 hours of *in vitro *culture. Images show normal expression pattern of ventral cell fate markers and proliferation marker Ki67. Shh and Olig2 (F-H), Pax6 and Ki67 (I-K), Nkx2.2 and Olig2 (L-N). Bar is 1.5 μm (A-C), 1 μm (D-E) and 50 μm (F-N).

In order to monitor cell divisions in the developing embryo, we optimized culture conditions for whole embryos and confirmed that morphological development and neural patterning proceeded normally over a 24-hour period in culture. First, we collected E7.5 embryos and cultured them for 24 hours (see Methods for details). We found these embryos were surrounded by their yolk sac and were morphologically indistinguishable from E8.5 embryos that developed *in utero *(Figure 3A, B, C). Second, we dissected E8.5 embryos and cultured them for 24 hours, at which time we found they had increased in size and their hearts initiated beating (n = 12 out of 13; Figure 3D, E). Additionally, we sectioned the neural tubes of these embryos and stained them for markers of ventral cell fates including Shh, Olig2, Pax6 and Nkx2.2, all of which looked normal (Figure 3F, G, H, I, J, K, L, M, N). Thus, our method of mouse embryo culture does not affect gross morphological development or influence the spatial distribution of developmental patterning markers. We next set out to image single cell divisions within the neuroepithelium under these culture conditions, which we call *ex vivo *imaging.

### Live imaging of single cell divisions within the neuroepithelium

Under the low-density conditions of cell culture, it was relatively easy to follow single cell divisions; however, the close packed cells of the neuroepithelium required us to label the cells. We used genetic lineage tracing since it is well established to indelibly label an individual cell and all subsequent progeny. We took advantage of two existing mouse lines: an ubiquitous tamoxifen-inducible Cre line, CAGGCreER™and a dsRed Cre reporter line, with a loxP-*βgeo*-STOP-pA-loxP cassette upstream of dsRed [13,14]. When these two lines are crossed, Cre-mediated recombination of the loxP sites excises the *βgeo*-STOP-pA, resulting in dsRed expression in all cell progeny. By injecting pregnant female mice with tamoxifen when the embryos were at E6.5, we induced Cre expression and dsRed labeling in a small subset of cells. This density of labeling enabled us to observe single cell divisions 48 hours later during *ex vivo *imaging.

**Figure 4:**
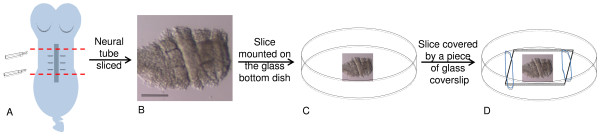
**Neural tube slice preparation for live imaging**. **(A) **Unturned embryo E8.5 with first appearance of somite pairs. **(B) **Neural tube dissected in pre-warmed medium using a micro-knife. **(C) **Slice of the neural tube mounted ventral side down on the poly-L-lysine coated glass bottom dish. **(D) **Small amount of a mixture made from petroleum jelly and wax applied around the neural tube and gently covered by a narrow piece of glass coverslip. Bar is 300 μm (B).

**Figure 5:**
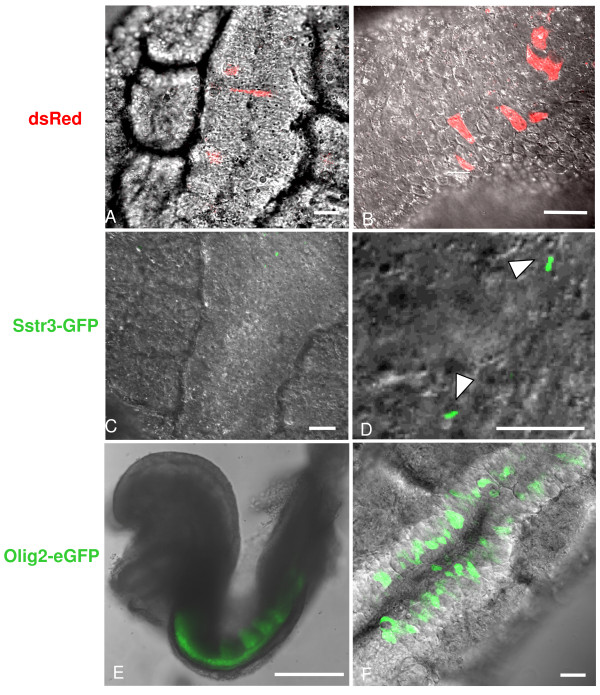
**Visualizing fluorescently tagged proteins in live cells of the neural tube with confocal microscopy**. **(A, B) **Low dose tamoxifen induces expression of DsRed at E8.5 in single cells within the embryo and neuroepithelium. **(C, D) **Cilia are marked by GFP in E8.5 embryo infected with SsTR3-GFP lentivirus. White arrowheads mark GFP expressed in cilia. **(E) **Sagittal view and **(F) **dorsal view of E8.5 embryo carrying Olig2-GFP shows GFP expression within neuroepithelium. Bar is 30 μm (A, B, C and F), 15 μm (D) and 1.5 μm (E).

In order to perform live imaging on the neuroepithelium, we harvested embryos at E8.5 and used a micro knife to slice the embryo in cross-sections. We mounted these on a glass bottom dish where we fashioned a small well using petroleum jelly and wax (Figure 4 and detailed in Methods). We placed culture media and the neural slice in this well before placing a coverslip on top. We then performed time-lapse confocal imaging to observe cell division, using the presence of dsRed to follow individual cells and their daughters (Figure 5A, B; Additional file 2).

### Live imaging of cilia formation and Shh response

Since Sstr3-GFP efficiently labeled cilia in cell culture and we had no mouse line containing a fluorescent cilia marker, we generated Sstr3-GFP lentivirus so that we could infect embryos in culture. When we infected the cultured embryos for approximately 18 hours prior to imaging, we could clearly observe cilia in the neuroepithelium (Figure 5C, D; Additional file 3).

**Figure 6:**
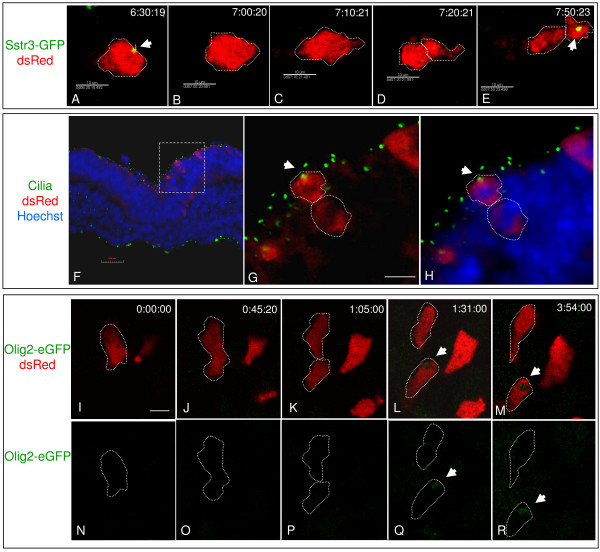
**Monitoring cilia formation and Sonic hedgehog signaling in dividing cells of the developing neural tube**. **(A-E) **Single dsRed positive cell undergoing division shows that a cilium forms in one daughter cell prior to the other cell (E, white arrow). The movie was imaged at a rate of one frame every 10 minutes. **(F-H) **Immunofluorescence using antibodies against red fluorescent protein in red (for dsRed lineage tracing) and Arl13b in green (for cilia) demonstrate that a cilium forms in one daughter cell prior to the other daughter cell (white arrow). Enlargement of boxed area in (F), without or with Hoechst staining (G, H). **(I-R) **The dsRed positive cell undergoes division (I-M). The Olig2-GFP is expressed only in one daughter (N-R). The recording was imaged at a rate of one frame every 10 minutes. Bar is 10 μm (A-E), 100 μm (F), 25 μm (G, H) and 5 μm (I-R).

The final tool we needed was a way to monitor the response to Shh signaling. Shh specifies the five ventral neural fates starting at E8.5 [10,11,15]. One domain, the progenitors of motor neuron cells, expresses the Shh-responsive transcription factor Olig2 [18-20]. The publically funded GENSAT project had previously modified a BAC containing Olig2 so that eGFP was inserted under the control of the Olig2 promoter, such that it would be Shh-responsive [21]. We obtained the cryopreserved line, generated mice, and found robust eGFP restricted to the Olig2-positive cells, albeit throughout the cytosol and not localized to the nucleus like endogenous Olig2 (Figures 5F and 6R). While the cytosolic eGFP often precluded us from observing cilia-localized Sstr3-GFP, the Olig2-eGFP line provided three advantages: it faithfully recapitulates the pattern of endogenous Olig2 expression; it expresses a robust level of GFP; and it responds to a level of Shh signaling we can easily see in the neuroepithelium [16]. Consequently, this line enabled us to monitor when a cell was Shh-responsive (Figure 5E, F; Additional file 4) [18].

### Asynchrony in cilia formation in dividing cells of the developing neural tube

**Figure 7:**
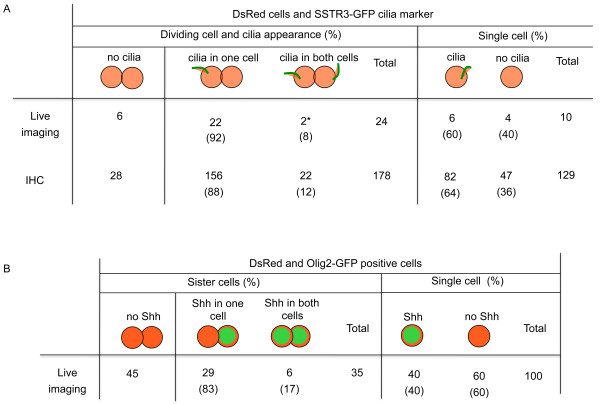
**Counting of dsRed positive cell with cilia and Sonic hedgehog appearance**. (A) Number of dsRed cells undergoing division and cilia localization. * by live imaging, cilia formation was synchronous. (B) Number of dsRed and Olig2-GFP positive cells undergoing division and Shh signaling.

To determine the relative timing of cilia formation in the daughter cells of an individual cell in the neuroepithelium, we integrated dsRed lineage tracing with the Sstr3-GFP lentivirus. We infected E8.5 tamoxifen-induced, dsRed Cre reporter embryos with Sstr3-GFP lentivirus during *in vitro *culture. After 18 hours, we sliced the embryo with the micro knife (Figure 4) and placed it on the glass bottom dish. Then we carried out time-lapse confocal imaging to observe dividing cells and their daughters. Of the 24 dsRed cell divisions with cilia appearance we watched, 92% displayed asynchrony of cilia formation in the daughter cells. In the remaining 8% of divisions during which one or more daughter cells expressed GFP, we saw sister cells simultaneously forming cilia (Figures 6A, B, C, D, E and 7A; Additional file 5).

In order to be sure that the Sstr3-GFP expression we were using to visualize cilia was not interfering with any underlying biological process, we confirmed these results in fixed samples that we stained with antibodies against dsRed and the ciliary protein, Arl13b [16]. After imaging 178 pairs of daughter cells in which at least one cell showed a cilium, we observed a single ciliated daughter cell in 88% of the pairs and dual ciliated daughter cells in 12% of the pairs (Figures 6F, G, H and 7A). Taken together, these data indicate that cilia formation between pairs of daughter cells is asynchronous in the developing mouse neural tube.

### Asynchrony in Shh signaling in daughter cells of the developing neural tube

To determine the relative timing of Shh response in the daughter cells within the neuroepithelium, we crossed the dsRed Cre reporter mice with the Olig2-eGFP BAC transgenic mice. After tamoxifen induction, we prepared samples from the dsRed Cre and Olig2-eGFP double positive embryos and recorded cell divisions under time-lapse confocal microscopy. We found that in 83% of pairs of daughter cells that responded to Shh, Olig2-eGFP was restricted to one daughter cell, and in 17% of such cell divisions it was in both daughter cells (Figures 6I, J, K, L, M, N, O, P, Q, R and 7B; Additional file 6). Thus, we conclude that the response to Shh is asynchronous between pairs of daughter cells in the neuroepithelium.

## Conclusions

Our results demonstrate that we can directly observe single cell divisions within the developing neuroepithelium and concomitantly monitor cilium formation or Shh response. We showed that when a cell divides in the developing mouse neural tube, both cilia formation and the Shh response are asynchronous between the resulting daughter cells. Thus, data generated using our *ex vivo *system are consistent with previous results from *in vitro *cultured cells [8]. This suggests that the experiments using cultured cells provide physiologically relevant data.

While we were unable to image cilia formation and Shh response in the exact same cell using our *ex vivo *method, three lines of evidence suggest that the daughter cell in which the cilium first forms is also the cell that first responds to Shh. First, the live imaging data show that, in the majority of cases, after a cell division only one cell has a cilium (92%) and only one cell displays a Shh-response (83%), making it likely that they are the same cell in most pairs (two-tailed *P *= 0.1695; degrees of freedom = 1). Second, cells without cilia cannot transduce Shh signaling [1,22,23]. Finally, our numbers are quite consistent with previous experiments in immortalized cell lines where the authors observed cilia formation and Shh signal transduction in the same cell [8]. Their study also demonstrated that the daughter cell that inherits the older mother centriole will be first to form a cilium and transduce a Shh response. Although our studies did not directly address this question, the consistency between their *in vitro *and our *in vivo *data would be consistent with the older centriole being inherited by the daughter cell that first forms a cilium and responds to Shh within the neuroepithelium as well. While formal proof will require further work, our method provides tools with which the field can more immediately monitor cell behavior. Coupled with the rich resources of mouse mutants, we expect this method to be especially powerful in examining whether cellular behavior can lead to both differentiation and maintenance of cells in a progenitor niche.

## Abbreviations

BAC: bacterial artificial chromosome; DMEM: (Dulbecco's) modified Eagle's medium; eGFP: enhanced GFP; FBS: fetal bovine serum; GFP: green fluorescent protein; Olig2: oligodendrocyte transcription factor 2; Pax6: paired box gene 6; Shh: Sonic hedgehog; SsTR3-GFP: somatostatin 3 receptor fused to GFP.

## Competing interests

The authors declare that they have no competing interests.

## Authors' contributions

KPN and TC designed the experiments and wrote the manuscript. KPN performed all experiments. All authors read and approved the final manuscript.

## Supplementary Material

Additional file 1 **Cilia formation in cultured cells: IMCD3 stably expressing SsTR3-GFP in cilia**. White arrows point to two dividing cells and follow daughter cell expressing SsTR3-GFP prior to the other daughter cell. Click here for file

Additional file 2 **Individual cell expressing dsRed undergoes division within the neuroepithelium**. White arrow indicates dividing dsRed cell. Click here for file

Additional file 3 **Neural tube infected with fluorescent cilia marker, SsTR3-GFP**. GFP expressed in cilia is pointed by the white arrow. Click here for file

Additional file 4 **Neural tube of the Olig2-GFP embryo expresses Shh in individual cells**. Two dividing cells that express Olig2-GFP are followed by the white arrow. Click here for file

Additional file 5 **Asynchrony in cilia formation in dividing dsRed cell infected with the SsTR-GFP lentivirus within the neuroepithelium**. GFP is expressed in cilia in one daughter cell following division and is pointed by the white arrow. Click here for file

Additional file 6 **Asynchrony in Shh signaling in daughter cells of the dsRed and Olig2BAC-GFP positive embryo within the neuroepithelium**. Olig2-eGFP is expressed in one daughter cell following division and is pointed by the white arrow. Click here for file
